# High-sensitive C-reactive protein and homocysteine levels in patients with newly diagnosed bipolar disorder, their first-degree relatives, and healthy control persons—Results from a clinical study

**DOI:** 10.1192/j.eurpsy.2020.105

**Published:** 2020-11-25

**Authors:** Marc Østergaard Nielsen, Nanna Aagaard Petersen, Klara Coello, Sharleny Stanislaus, Sigurd A. Melbye, Hanne Lie Kjærstad, Kimie Stefanie Ormstrup Sletved, Ruth Frikke-Schmidt, Roger S. McIntyre, Maj Vinberg, Lars Vedel Kessing

**Affiliations:** 1 Copenhagen Affective Disorders Research Centre (CADIC), Psychiatric Centre Copenhagen, Rigshospitalet, Copenhagen, Denmark; 2 Department of Clinical Medicine, Faculty of Health and Medical Sciences, University of Copenhagen, Copenhagen, Denmark; 3 Department of Clinical Biochemistry, Centre of Diagnostic Investigation, Rigshospitalet, Copenhagen, Denmark; 4 Mood Disorders Psychopharmacology Unit, University Health Network, Toronto, Ontario, Canada; 5 Psychiatric Research Unit, Psychiatric Centre North Zealand, Hillerød, Denmark

**Keywords:** Bipolar disorder, homocysteine, C-reactive protein, unaffected relatives

## Abstract

**Background:**

Changes in inflammatory and metabolic markers are implicated in the pathogenesis in both the development and progression of bipolar disorder (BD). Notwithstanding, these markers have not been investigated in newly diagnosed BD.

**Methods:**

We compared high-sensitive C-reactive protein (hs-CRP) and homocysteine (Hcy) levels in 372 patients with newly diagnosed BD, 106 unaffected first-degree relatives (URs), and 201 healthy control persons (HCs). Within the patient group, we also investigated possible associations between hs-CRP and Hcy, respectively, with illness-related characteristics and psychotropic medication.

**Results:**

No statistically significant differences in Hcy and hs-CRP levels were found when comparing BD and URs with HCs. Similarly, there were no differences when comparing only patients in remission or patients with affective symptoms, respectively, with HCs. Hcy levels were found to be 11.9% (95% CI: 1.030–1.219) higher in patients with BD when compared with their URs (*p* = 0.008), when adjusting for folate and cobalamin status, age, sex, and self-reported activity levels. Hcy levels were significantly associated with folate, cobalamin, gender, and age in all models (*p* < 0.05).

**Conclusion:**

Our results do not support hs-CRP or Hcy as markers in newly diagnosed BD.

## Introduction

Bipolar disorder (BD) is often a progressive disorder with an increasing risk of recurrence after each depressive and manic episode [1]. Patients with BD often experience functional disability, decreased quality of life, and an overall reduced life expectancy of 8–12 years [2]. BD is associated with biochemical evidence of increased inflammatory burden especially in the acute mood states [3], suggesting that inflammation is involved in the underlying pathophysiology possibly regarding both the onset and the progression of the disease [4,5].

C-reactive protein (CRP) is an acute-phase protein but may also be increased outside the acute phase, and CRP levels rise in response to inflammatory stimuli primarily induced by interleukin-6 and interleukin-1β cytokines [6]. High levels of CRP are frequently studied in BD and overall seem to constitute a promising biomarker in BD (reviewed in [3]).

More recently, homocysteine (Hcy) has gained particular attention [7], since alterations in the one-carbon metabolism might be implicated in psychiatric disorders [8,9]. Hcy is a nonprotein-forming sulfurated amino acid, derived from the essential amino acid “methionine.” Methionine is primarily ingested through animal foods, such as beef, fish, dairy products, and eggs, though high levels can also be found in Brazil nuts and sesame seeds [10].

The metabolism of Hcy involves two pathways: remethylation to methionine and trans-sulfuration to cystathionine, which is further metabolized into cysteine [11]. Both pathways are dependent on multiple cofactors and enzymes; the most investigated being methylenetetrahydrofolate reductase (MTHFR), and the cofactors vitamin B12, vitamin B6, and folate [12]. Consequently, nutritional deficiencies in either vitamin B12 or folate and reduced activity of MTHFR may lead to hyperhomocysteinemia [13,14].

Hyperhomocysteinemia is associated with neuro- and vasculotoxic effects [15] caused by several proposed mechanisms such as induction of oxidative stress [8], neuroapoptosis [16], inflammation [17], direct vascular damage [18], aberrant DNA methylation[19], and impaired DNA synthesis [20]. Furthermore, hyperhomocysteinemia is a known risk factor for cardiovascular diseases [21], Alzheimer’s disorders [22], schizophrenia [9,19], depression [23,24], and autism spectrum disorders [25].

The first meta-analysis to assess Hcy as a possible biomarker for BD included 10 articles comprised of 663 patients with BD and 884 HCs. The researchers found elevated Hcy levels in both euthymic and manic states when compared with healthy controls [26]. While the study was able to shed light on the proposed mechanisms for Hcy’s effect in the development and progression of BD, it did not asses high-risk individuals such as first-degree relatives to patients with BD or consider the duration of illness.

In summary, hs-CRP and Hcy levels potentially play a role in the pathogenesis of BD. However, whether the two biomarkers act as state or trait (being present prior to and following onset of the illness and potentially increasing with clinical progression of the illness [1]) markers is not clarified. Finally, there is a lack of studies exploring hs-CRP and Hcy levels in newly diagnosed BD and their unaffected first-degree relatives (URs).

The aim of the present study was therefore to investigate high-sensitive CRP and Hcy levels in patients with newly diagnosed/first-episode BD, their URs, and HCs without a family history of psychiatric disorders (HC).

We hypothesize that hs-CRP and Hcy levels are elevated in patients with BD and—to a lesser degree—in their URs when compared to healthy individuals without a family history of psychiatric disorders. Furthermore, we hypothesize that hs-CRP and Hcy levels are associated with severity of depressive and manic symptoms and with longer illness duration.

## Methods

### Study design

The present study is a cross-sectional investigation of baseline data from the ongoing, longitudinal Bipolar Illness Onset (BIO) study, which aims to identify composite biomarkers for BD. A full research protocol has been published for the BIO cohort study [27]. Recruitment in the BIO study started in June 2015 and ended in November 2019.

### Participants

#### Patients with bipolar disorder

Patients were recruited in the Copenhagen Affective Disorder Clinic, which covers the entire greater Copenhagen catchment area (Region Hovedstaden). The Copenhagen Affective Disorder Clinic provides treatment services for patients with newly diagnosed BD and receives patients from the entire Capital Region of Denmark covering a catchment area of 1.6 million people and all psychiatric centers in the region. All patients, aged 18–70 years, referred to the Copenhagen Affective Disorder Clinic as patients with newly diagnosed BD, that is, onset of first manic or hypomanic episode or when the diagnosis of BD is made for the first time, were routinely asked for inclusion in the BIO study. Exclusion criteria were having BD secondary to brain injury.

#### Unaffected first-degree relatives

Siblings and children, aged 18–70 years, to the included patients with BD were, after consent, invited to participate in the BIO study. Siblings or offspring diagnosed with BD or schizophrenia, half-siblings, and adopted children/siblings were not included.

#### Healthy control persons

HCs were recruited among blood donors from the Blood Bank at Rigshospitalet Copenhagen, Denmark. The donors were approached in the waiting room on random days and invited to participate if they met the inclusion criteria, aged between 18 and 70 years and no personal or first-degree history of psychiatric disorder that required treatment.

### Diagnostic assessment and data collection

Diagnostic assessments including current affective states of the included patients were performed by medical doctors specialized in psychiatry according to the ICD-10 and DSM criteria for type I and type II BDs.

After informed consent, medicine or psychology Ph.D. students verified the diagnosis utilizing the Schedules for Clinical Assessment in Neuropsychiatry (SCAN) [28]. Furthermore, the clinical assessments of severity of depressive and manic symptoms were done using the Hamilton Depression Scale-17 (HAMD-17) items [29] and the Young Mania Rating Scale (YMRS) [30], respectively. Medication, alcohol intake, and smoking habits were recorded, and activity levels and sleep patterns were recorded using the following questionnaires.

The Pittsburgh Sleep Quality Index (PSQI) [31] is a self-rated questionnaire, consisting of 19 items, which generate seven component scores resulting in a global sleep quality score. The component scores cover areas such as subjective sleep quality, sleep latency, sleep duration, and sleep disturbances. A global score greater than 5 indicates poor sleep quality.

The Copenhagen City Heart Study (CCHS) physical activity questionnaire [32] was used to assess physical activity, including four categories ranging from less than 2 h of light activity per week to more than 4 h of strenuous activity per week.

Height and weight were measured to nearest centimeter and 0.1 kg, respectively, with the participant being lightly dressed and without shoes.

### Blood sample collection and analysis

Fasting blood samples (including hs-CRP and Hcy) were collected in a resting state between 7.30 AM and 10 AM the same day as the clinical assessment. Blood sampling and all aspects of laboratory processing were done at the Department of Clinical Biochemistry, Rigshospitalet, by laboratory specialists blinded for participant status.

For all samples, blood was drawn by venipuncture into a vacuum tube containing either lithium heparin (Hcy and hs-CRP) or EDTA (folate and cobalamin) and was, within 1 h after delivery, centrifuged at 2000 g at ambient temperature (Hcy and hs-CRP) or 4°C (folate and cobalamin) for 10 min.

After separation, Hcy concentrations were determined in plasma by spectrophotometry on a Konelab 30i (Thermo Scientific, Vantaa, Finland) random access clinical chemistry system using reagents and calibrators from DiaSys.

CRP (hs-CRP) was measured in plasma using a latex immunoturbidimetric assay (LIA) via the Cobas 8000 biochemistry analyzer, Roche Diagnostic, Meylan, France.

Folate and cobalamin levels were measured by electrochemiluminescence immunoassay (ECLIA) on the Cobas 8000 module e801.

The measuring range for the assays were: 4.5–45.4 nmol/L (folate); 111–1480 pmol/L (cobalamin); 1.0–150 μmol/L (Hcy); and 0.3–20 mg/L (hs-CRP).

### Statistical analyses

Categorical descriptive data were analyzed using the chi-squared test. Continuous descriptive data were first explored for homogeneity of variance to determine whether a nonparametric or parametric test should be utilized in the multiple group comparisons. If the data were parametric, a one-way ANOVA with post hoc Tukey’s test was used for the comparisons. If the data were nonparametric, it was described as medians and quartiles and compared between groups with a Kruskal–Wallis test followed by a Dunn–Bonferroni post hoc test for the pairwise comparisons.

Data include dependent measures between patients with BD and their relatives and were analyzed using generalized linear mixed models as done in our previous studies from the BIO study comparing patients with BD and their URs with healthy individuals [33,34]. In these linear mixed-effect models, family relationship was included as a random effect to account for the correlation between family-related individuals. The analysis strategy was planned a priori.

To obtain a normal distribution of Hcy, the values were log-transformed and presented as back-transformed values, estimate *B*, representing the relative mean difference between groups. Model 1 was unadjusted for any covariates, model 2 was adjusted for folate and cobalamin concentrations, model 3 was additionally adjusted for age and sex, and finally model 4 was further adjusted for self-reported activity levels.

Finally, subanalyses within patients were conducted using multiple linear regression to comprise associations of hs-CRP and Hcy, respectively, with psychotropic medication status, assessed as four categorical variables (yes/no) with regards to either lithium, antidepressants, antipsychotics, or antiepileptics. Some patients received no medications at time of assessment, leading to the creation of a fifth categorical variable, medication free versus receiving psychotropic medication. Illness-related variables were also explored: duration of illness, age at first affective episode, delay in diagnosis (years), and HAMD-17 or YMRS total score.

Secondary illness-related variables were explored in post hoc analyses. All statistical analyses were performed using SPSS version 25 (SPSS for Windows Inc., Chicago, IL), and the level of significance was set at *p* < 0.05.

### Ethics

The study protocol was approved by the Committee on Health Research Ethics of the Capital Region of Denmark (Protocol No. H-7-2014-007) and the Danish Data Protection Agency of the Capital Region of Copenhagen (RHP-2015-023). Written informed consent was provided by all participants. The study complied with the Declaration of Helsinki principles.

## Results

### Demographic and clinical characteristics

We included 679 participants comprised of 372 patients newly diagnosed with BD, 106 URs, and 201 HCs.

For the present analyses, 19 patients with BD, 5 URs, and 1 HC were excluded due to missing blood samples.

Demographic and clinical characteristics of the participants are presented in Table 1.

**Table 1.** Demographic, clinical characteristics, medication, and plasma homocysteine (Hcy) and high-sensitive C-reactive protein (hs-CRP) levels in patients with bipolar disorder (BD)^1^, their unaffected relatives (URs)^2^, and healthy control persons (HCs)^3^.

	BD^1^	UR^2^	HC^3^	*p* value
*N*	372	106	201	
Age (years)	29 [24.2–36.8]	26.7 [22.6–31.9]	27.3 [24.3–36]	0.005^1–2^ 1.000^1–3^ 0.071^2–3^
Female sex, *N* (%)	242 (65.1)	60 (56.6)	129 (64.2)	0.273*
Education (year’s total)	15 [12.5–17]	15 [13–17]	16 [14.5–17]	1.000^1–2^ <0.001^1–3^ 0.025^2–3^
HAMD-17 (total score)	9 [5–15]	0 [2–4]	0 [0–2]	<0.001^1–2^ <0.001^1–3^ 0.002^2–3^
YMRS (total score)	3 [0–7]	0 [0–2]	0 [0–1]	<0.001^1–2^ <0.001^1–3^ 1.000^2–3^
BMI	24.56 [22.11–27.31]	23.85 [21.26–26.88]	23.69 [21.88–26.25]	0.079*
Number of current smokers (%)	160 (43.7)	21 (21.6)	22 (10.9)	<0.001^1–2^ <0.001^1–3^ 0.181^2–3^
Alcohol consumption (weekly)	4.7 ± 6.8 (0–42)	4.6 ± 5.4 (0–22)	6.2 ± 5.5 (0–36)	0.980^1–2^ 0.016^1–3^ 0.075^2–3^
Age of onset	22.6 ± 8.6 (4–60) 20 [17–26]	–	–	
Illness duration (years)**	11.6 ± 8.5 (0–45)	–	–	
Untreated BD (years)***	7.1 ± 8.0 (−1 to 39) 4 [1–10.5]	–	–	
BD I/BD II (%)	118/254 (31.7/68.3)	–	–	
Current affective state				
Remission (HAMD-17 and YMRS < 8)	215 (58.4)	–	–	
Depressive episode mild/moderate (HAMD-17 < 25)	90 (24.5)	–	–	
Severe depressive episode (HAMD-17 ≥ 25)	10 (2.7)	–	–	
Manic episode (YMRS > 16)	1 (0.3)	–	–	
Hypomanic episode (YMRS < 16)	31 (8.4)	–	–	
Mixed episode (HAMD-17 > 13 and YOUNG > 8)	19 (5.2)	–	–	
N.A.	2 (0.5)	–	–	
Current psychotropic medication****				
No psychotropic medication	65 (17.5)	–	–	
Antidepressant treatment	47 (12.6)	–	–	
Antipsychotic treatment	135 (36.3)	–	–	
Antiepileptic treatment	193 (51.9)	–	–	
Lithium treatment	112 (30.1)	–	–	
p-Hcy levels (μmol/L)	*N* = 366 13.29 ± 6.30 (12.64–13.94) 12.3 [9.48–15.93]	*N* = 101 11.93 ± 4.15 (11.11–12.75) 11.5 [9–14.5]	*N* = 200 12.65 ± 4.97 (11.96–13.34) 11.8 [9.5–14.8]	0.081^1–2^ 0.397^1–3^ 0.550^2–3^
p-hs-CRP (mg/L)	1.67 ± 2.52 (1.42–1.93) *N* = 373	1.66 ± 2.55 (1.17–2.15) *N* = 106	1.68 ± 2.79 (1.29–2.06) *N* = 201	

Notes: Data are presented as mean (SD ± range), median [interquartiles], or *N* (%) unless otherwise stated.

Abbreviations: HAMD-17, 17-item Hamilton Depression Rating Scale; YMRS, Young Mania Rating Scale; BD I and BD II, bipolar disorder type I and II, respectively; N.A, not available; BMI, body mass index.

* Multiple comparisons are not performed because the overall test does not show significant differences across samples.

** Illness duration defined as time from first mood episode to inclusion date.

*** Untreated BD defined as time from first mood episode to time of diagnosis.

**** The percentages of patients receiving psychotropic medication exceed 100% as several patients with BD received more than one medication at the time of inclusion.

No significant differences in height, weight, gender distribution, or body mass index (BMI) were observed between the groups. The UR group was on average younger than the patient group (*p* < 0.05). The HC group consumed more alcohol per week compared with the patient group (*p* = 0.02) and had statistically higher education levels compared with both the patient (*p* < 0.01) and UR groups (*p* = 0.03). Finally, more patients with BD were smokers compared with the groups of UR and HC, respectively.

### Plasma homocysteine levels in patients with newly diagnosed bipolar disorder, their unaffected first-degree relatives, and healthy control persons

**Figure 1.**
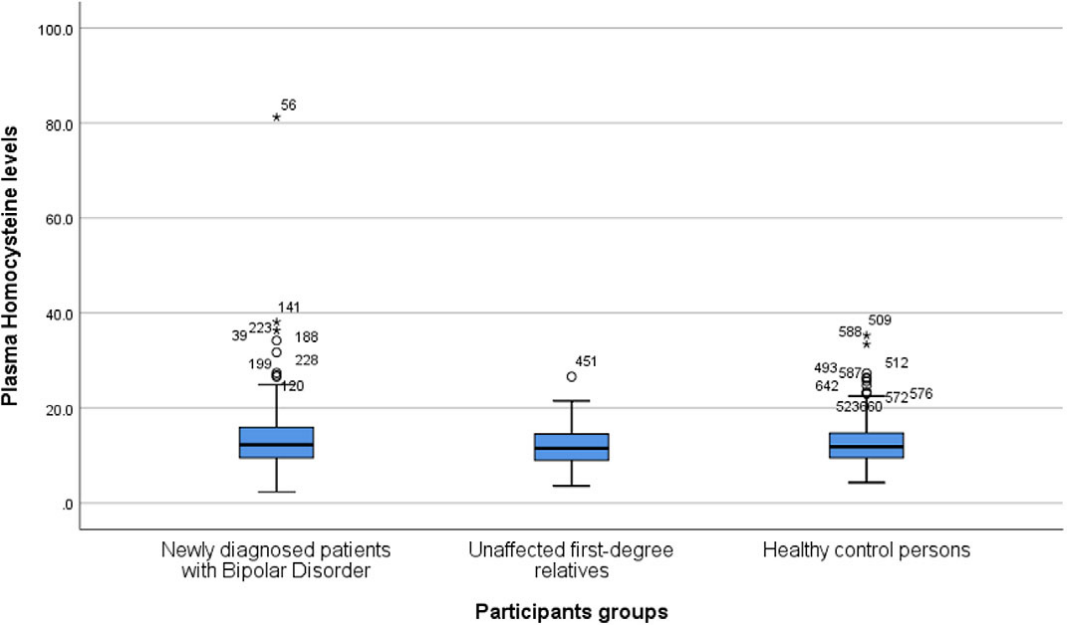
Boxplot depicting homocysteine levels (micromole per liter) in patients newly diagnosed with bipolar disorder, their unaffected first-degree relatives, and healthy control persons. The lower and upper hinges represent the first and third quartiles, and the upper and lower whiskers extend from the hinge to the largest and lowest values, correspondingly. Data beyond the end of the whiskers are plotted individually.

As can be seen from Figure 1 and Table 2, no significant differences in Hcy levels were found when comparing patients newly diagnosed with BD or their URs to HCs in all models. Furthermore, when only patients in remission (HAMD-17 < 8 and YMRS < 8) were included, no differences were found between patients (*N* = 116) and HCs (*N* = 198; *p* = 0.4). Similarly, when only patients with affective symptoms/episodes (HAMD-17 ≥ 8 and YMRS ≥ 8) were included, no differences were found between patients (*N* = 250) and HCs (*N* = 200; *p* = 0.4).

**Table 2.** Homocysteine levels in patients newly diagnosed with bipolar disorder (BD), their unaffected first-degree relatives (URs), and healthy control persons (HCs).

	*B*	95% CI	*p* value
Model 1*			
BD vs. UR	1.085	0.997–1.180	0.058
BD vs. HC	1.035	0.968–1.107	0.317
UR vs. HC	0.954	0.868–1.048	0.327
Model 2**			
BD vs. UR	1.121	1.033–1.216	0.007
BD vs. HC	1.036	0.970–1.105	0.293
UR vs. HC	0.924	0.842–1.014	0.095
Folate	0.985	0.981–0.988	<0.001
Cobalamin	0.999	0.999–0.999	0.007
Model 3***			
BD vs. UR	1.109	1.024–1.201	0.011
BD vs. HC	1.043	0.979–1.110	0.191
UR vs. HC	0.940	0.860–1.029	0.178
Folate	0.986	0.983–0.989	<0.001
Cobalamin	0.999	0.999–0.999	<0.001
Age	1.006	1.003–1.009	<0.001
Male vs. female	1.180	1.111–1.253	<0.001
Model 4****			
BD vs. UR	1.119	1.030–1.219	0.008
BD vs. HC	1.047	0.977–1.119	0.187
UR vs. HC	0.933	0.851–1.026	0.154
Folate	0.986	0.983–0.990	<0.001
Cobalamin	0.999	0.999–0.999	<0.001
Age	1.005	1.002–1.008	0.001
Male vs. female	1.172	1.100–1.249	<0.001
Total CCHS*****	1.023	0.983–1.064	0.265

* *N*: BD = 366, UR = 101, and HC = 200.

** *N*: BD = 325, UR = 90, and HC = 190.

*** *N*: BD = 325, UR = 90, and HC = 190.

**** *N*: BD = 295, UR = 84, and HC = 175.

***** CCHS: Copenhagen City Heart Study—self-reported activity levels.

In the unadjusted model 1, patients exhibited a trend toward having higher levels of Hcy than URs (*B* = 1.085, 95% CI: 0.997–1.180, and *p* = 0.058). In addition, in the adjusted models 2–4, this comparison yielded statistical significance (*p* < 0.05). Folate, cobalamin, age, and gender had statically significant associations with Hcy levels in all between-group analyses (*p* < 0.05). In the final fully adjusted model 4, we found patients with BD to have 11.9% increased Hcy levels compared with their URs (*B* = 1.119, 95% CI: 1.030–1.219, and *p* = 0.008). In the same model, participants with BD had 4.7% (*B* = 1.047 and 95% CI: 0.977–1.119) higher levels of Hcy compared with HCs, while URs had 6.7% lower Hcy levels compared with HCs; however, these were not statistically significant (*p* > 0.05).

### Plasma hs-CRP levels in patients with newly diagnosed bipolar disorder, their unaffected first-degree relatives, and healthy control persons

As shown in Table 3, no significant differences in hs-CRP levels were found between the groups; moreover, the mean concentrations of the three groups were nearly identical with *p* values approaching 1. Further adjustment for age and sex did not alter results. In addition, there was no difference when only patients in remission (HAMD-17 < 8 and YMRS < 8) were included between patients (*N* = 118) and HCs (*N* = 199; *p* = 0.2). Similarly, when only patients with affective symptoms/episodes (HAMD-17 ≥ 8 and YMRS ≥ 8) were included, no differences were found between patients (*N* = 254) and HCs (*N* = 201; *p* = 0.4).

**Table 3.** High-sensitive C-reactive protein levels in patients newly diagnosed with bipolar disorder (BD), their unaffected first-degree relatives (URs), and healthy control persons (HCs).

Model 1*	Mean difference	95% CI	*p* value
BD vs. UR	−0.008	−0.564 to 0.548	0.977
BD vs. HC	−0.003	−0.451 to 0.446	0.990
UR vs. HC	0.005	−0.613 to 0.624	0.986

* *N*: BD = 372, UR = 106, and HC = 201.

### Associations between illness-related variables and psychotropic medication status in patients with newly diagnosed bipolar disorder

No associations were found between duration of illness, age at first manic episode, and delay in diagnosis with regards to hs-CRP levels, and only age at first hypomanic or manic episode was associated with Hcy levels, however, with small effects (*B* = 0.67%, *p* > 0.01, and *R*^2^ = 0.02).

Moreover, multiple linear regression analysis revealed no significant associations of psychotropic medication status with either Hcy or hs-CRP levels.

### Post hoc explorative analysis

Within the patient group, we explored the following secondary illness-related variables: age, gender, sleep (PSQI total score), physical activity levels (CCHS), smoking status, BMI, HAMD-17 and YMRS total scores, folate and cobalamin levels, and lastly alcohol intake—with multiple linear regression, resulting in a model (adjusted *R*^2^ = 0.239) with significant associations of Hcy levels with age (*p* = 0.021), gender (*p* = 0.004), YMRS total score (*p* = 0.029), folate (*p* > 0.001), and cobalamin levels (*p* = 0.004). As Hcy values were log-transformed, the *B* values for the abovementioned variables have been transformed to the following relative differences: compared with women, men were found to have 15% higher levels of Hcy, age was associated with increasing Hcy levels by 0.57% per year, and for every point in YMRS, Hcy levels increased by approximately 1%. Folate decreased Hcy levels by 1.5% per unit, and for every 100 units of cobalamin, Hcy levels decreased by approximately 4.6%.

Utilizing the same method for hs-CRP levels resulted in a model (adjusted *R*^2^ = 0.121) with significant associations to BMI (*B* = 0.128 and *p* < 0.001), PSQI (*B* = 0.081 and *p* = 0.009), CCHS (*B* = −0.39 and *p* = 0.017), and lastly alcohol over/under 14 units per week (*B* = 0.95 and *p* = 0.044).

There were no differences in Hcy (*p* = 0.9) or hs-CRP(*p* = 0.07) levels between patients with BD type I and II.

## Discussion

The BIO study is the first to investigate Hcy and hs-CRP levels in newly diagnosed patients with BD and their URs in comparison to HCs.

No statistically significant differences were found in Hcy or hs-CRP levels in patients with newly diagnosed BD or their first-degree URs compared with healthy controls. We did however find statistically significant higher levels of Hcy in patients with BD compared with their first-degree URs, and we found Hcy levels significantly correlated with cobalamin, folate, age, and sex in all models.

### Interpretation of findings

Our results do not support hs-CRP or Hcy as markers in newly diagnosed BD.

The negative findings could be attributed rapid diagnostic clarification and subsequent treatment as we included patients referred to a specialized mood disorder clinic, the Copenhagen Affective Disorder Research Center (CADIC).

The included patients had a median age of 20 years (quartiles: 24.2–36.8) at illness onset and a median delay in diagnosis of 4 years. Most patients received at least one psychotropic medication, were euthymic at the time of inclusion, and had a significantly lower weekly consumption of alcohol when compared with the healthy control group (*p* = 0.016).

These findings could be interpreted as a successful influence of the early intervention and treatment all patients receive at the specialized mood disorder clinic, CADIC. The treatment includes a focus on psychiatric medication and group-based psychoeducation including the importance of abstaining from alcohol. A previous study found that patients treated at this outpatient clinic used mood stabilizers more often and had a significant decrease in readmissions compared with patients receiving standard treatment [35].

#### High-sensitive C-reactive protein

The replicated finding of elevated hs-CRP in samples of adults with BD [3,36] largely represents individuals with multiepisode and/or later stage BD, with a paucity of studies conducted in persons early in the illness trajectory. Moreover, individuals with multiepisode and later stage BD are more likely to have comorbid conditions and behaviors (e.g., obesity) that are associated with a proinflammatory balance. In addition, CRP levels may, to a greater extent, act as a state factor as we observed in a previous study from our group [37].

It has been proposed that certain psychotropic medications (e.g., olanzapine, quetiapine, lithium, and divalproex) possess immunosuppressive properties [38], by downregulating proinflammatory mRNA and subsequently protein gene expression, possibly leading to lower levels of cytokines resulting in lower hs-CRP levels. On the other hand, the aforementioned psychotropic medications are also associated with weight gain, and CRP levels have been positively correlated with waist circumference and diastolic blood pressure in euthymic patients with BD [39]. Patients with BD tend to live a more sedentary lifestyle compared with healthy controls [40] and subsequently have a higher prevalence of obesity [41]. However, we could not, in this newly diagnosed group of patients with BD, replicate this finding as we found no significant differences in BMI between the groups.

#### Homocysteine

The findings from our study may suggest patients with BD have higher levels of Hcy compared with their URs when age, gender, or folate and cobalamin status are considered; however, this finding might also be a random secondary finding, since it was not seen in the unadjusted model.

Higher age and male sex were significantly associated with higher levels of Hcy across all groups in the present study. Healthy male donors have shown to have higher mean levels of Hcy compared with healthy female donors [42]. This gender correlated difference is thought to be mediated by estrogens, which seemingly possess an inverse correlation with Hcy [43].

Lower levels of folate and cobalamin were significantly associated with higher levels of Hcy across all groups. This is in line with findings from other studies [42,44].

However, the patients with BD included here comprise a much younger population than other studies investigating Hcy levels in patients with BD. For example, in eight of the nine studies included in the meta-analysis by Salagre *et al.* [26], the mean population age of patients with BD varied from 37.8 to 49.8 years in contrast to a mean age of 31.3 years in the present study. In addition, in six studies from the meta-analysis, duration of illness mean years varied from 10.95 to 16.7 years compared with 11.6 years in the present study. Notably, in the same meta-analysis, no relationship with Hcy and neither age nor sex was found.

### Study limitations and strengths

The design of the BIO study offers several advantages. First, all patients with newly diagnosed BD in the BIO study were initially diagnosed by a clinical psychiatrist before being verified by trained medicine or psychology Ph.D. students, utilizing the semi-structured SCAN interview. Second, all blood sampling and subsequent analyses were carried out following standardized operating procedures and the laboratory staff was blinded to participant status. Third, with a median age of illness onset of 20 years and a median delay in diagnosis of 4 years, the population with BD in the BIO study is representative of patients newly diagnosed with BD, which in turn makes it possible to investigate variables unaffected by long-term illness [45,46].

The sample of healthy controls included in this study was recruited from the Blood Bank at Rigshospitalet, Copenhagen. This population may be vulnerable to a healthy donor effect, in which blood donors may have lower rates of mortality and morbidity, compared with the general population [47]. Nevertheless, the blood donors included in this study were recruited from the same catchment area as patients with BD, they did not differ in sex composition and only slightly in age and educational level from patients, and they were not granted economic compensation for participating. Alternative methods for recruiting control groups include using advertisements or the Danish Civil Registration System. However, both these methods have relatively low participation response rates and a high risk of selection bias. Taken together, we find that our control group represents the most reasonable and accessible control group for this study.

One of the main limitations of our study may be the lack of dietary information about our participants. The field of nutritional psychiatry has amassed a significant body of evidence, supporting the theory that dietary patterns have a considerable relationship with common mental illnesses [48]; moreover, a study from our group showed the gut microbiota of patients with BD differed from healthy controls [49].

While we did incorporate folate and cobalamin levels in our statistical analyses of Hcy, we could not determine whether folate or cobalamin deficiencies are caused by nutritional deficiencies, psychotropic medication, or BD itself.

Furthermore, we are limited by our information regarding exercise patterns of our participants. We utilized a self-reported activity questionnaire, in which participants selected one of four statements that best described their general activity levels. Self-reported activity levels in patients with BD have shown to be higher than objectively assessed levels [40].

The majority of patients included in the analyses were in full or partly remission (HAMD and Young < 17), and only 10 patients had a severe depression and 1 patient a manic episode (Table 1). Consequently, we cannot exclude the possibility that Hcy or hs-CRP may be increased during severe depressive or manic episodes among patients with newly diagnosed BD.

Finally, although there were no differences in hs-CRP or Hcy levels between patients with BD type I and II and although the sample included 118 patients with BD type I, it cannot be excluded that inclusion of more patients with type I BD would have led to other results.

## Conclusion

Overall, our results do not support hs-CRP or Hcy as markers in newly diagnosed BD. These findings could be a result of successful early intervention and treatment as offered in a specialized mood disorder clinic including psychiatric medication and group-based psychoeducation.

## Data Availability

Data are not available.
